# Paradoxal Trends in Azole-Resistant *Aspergillus fumigatus* in a National Multicenter Surveillance Program, the Netherlands, 2013–2018

**DOI:** 10.3201/eid2607.200088

**Published:** 2020-07

**Authors:** Pieter P.A. Lestrade, Jochem B. Buil, Martha T. van der Beek, Ed J. Kuijper, Karin van Dijk, Greetje A. Kampinga, Bart J.A. Rijnders, Alieke G. Vonk, Sabine C. de Greeff, Annelot F. Schoffelen, Jaap van Dissel, Jacques F. Meis, Willem J.G. Melchers, Paul E. Verweij

**Affiliations:** VieCuri Hospital, Venlo, the Netherlands (P.P.A. Lestrade);; Center of Expertise in Mycology Radboud University Medical Center/Canisius-Wilhelmina Hospital, Nijmegen, the Netherlands (J.B. Buil, J.F. Meis, W.J.G. Melchers, P.E. Verweij);; Leiden University Medical Center, Leiden, the Netherlands (M.T. van der Beek, E.J. Kuijper);; Amsterdam University Medical Center, Vrije Universiteit Amsterdam, Amsterdam, the Netherlands (K. van Dijk);; University of Groningen, University Medical Center Groningen, Groningen, the Netherlands (G.A. Kampinga);; Erasmus Medical Center, Rotterdam, the Netherlands (B.J.A. Rijnders, A.G. Vonk);; Center for Infectious Disease Control, Dutch National Institute for Public Health and the Environment, Bilthoven, the Netherlands (S.C. de Greeff, A.F. Schoffelen, J. van Dissel)

**Keywords:** Aspergillus fumigatus, antimicrobial resistance, azole-resistant, surveillance, fungal infections, the Netherlands, fungi

## Abstract

We investigated the prevalence of azole resistance of *Aspergillus fumigatus* isolates in the Netherlands by screening clinical *A. fumigatus* isolates for azole resistance during 2013–2018. We analyzed azole-resistant isolates phenotypically by in vitro susceptibility testing and for the presence of resistance mutations in the *Cyp51A* gene. Over the 6-year period, 508 (11%) of 4,496 culture-positive patients harbored an azole-resistant isolate. Resistance frequency increased from 7.6% (95% CI 5.9%–9.8%) in 2013 (58/760 patients) to 14.7% (95% CI 12.3%–17.4%) in 2018 (112/764 patients) (p = 0.0001). TR_34_/L98H (69%) and TR_46_/Y121F/T289A (17%) accounted for 86% of Cyp51A mutations. However, the mean voriconazole MIC of TR_34_/L98H isolates decreased from 8 mg/L (2013) to 2 mg/L (2018), and the voriconazole-resistance frequency was 34% lower in 2018 than in 2013 (p = 0.0001). Our survey showed changing azole phenotypes in TR_34_/L98H isolates, which hampers the use of current PCR-based resistance tests.

*Aspergillus fumigatus* is a saprobic mold that thrives on decaying plant material. The fungus is thermotolerant and exhibits optimum growth at 37°C. *A. fumigatus* has evolved into an major cause of pulmonary infections, especially in immunocompromised persons. Patients at risk for invasive aspergillosis include patients with hematologic malignancy, solid organ transplant recipients, and patients receiving corticosteroids. In addition, new risk groups are being recognized, including patients treated with ibrutinib (*1*) and patients with severe influenza (*2*,*3*). The fungus might also cause chronic pulmonary infections, chronic lung colonization, and allergic syndromes (*4*). 

Azoles represent the most important class of antifungal agents that are used for the management of *Aspergillus* diseases. Triazoles with activity against aspergilli include itraconazole, voriconazole, posaconazole, and isavuconazole. However, use of this drug class has been threatened by the emergence of azole resistance, which was first reported in 1997 (*5*). Although resistance might be selected during azole therapy, resistance selection in the environment through exposure to azole fungicides has been shown to be the most important route for resistance selection (*6*). The environmental route of resistance selection poses numerous challenges for patient management because two thirds of patients with azole-resistant invasive aspergillosis have no previous history of azole therapy (*7*). A recent cohort study showed that voriconazole resistance resulted in 21% lower day-42 survival in patients with culture-positive invasive aspergillosis compared with voriconazole-susceptible infection, indicating a major effect of resistance on patient survival (*8*). Because most patients with invasive aspergillosis are culture negative, sensitive non–culture-based resistance tests are urgently needed (*9*).

Although azole-resistant *A. fumigatus* was recently added to the antibiotic threats list of the US Centers for Disease Control and Prevention (*10*), systematic resistance surveillance programs are currently lacking. Surveillance is hampered by low numbers of culture-positive patients, difficulty in diagnosing and classifying patients with *Aspergillus* diseases, low awareness of fungal resistance, and limited prioritization of fungal resistance research. Furthermore, unlike bacteria, molds do not routinely undergo resistance testing in most clinical microbiology laboratories, thus necessitating the implementation of specific laboratory protocols. Early reports on azole resistance in the Netherlands prompted the national Center for Infectious Disease Control (Cib) to support a reference laboratory to set up a surveillance network to monitor trends in azole-resistance frequency in *A. fumigatus*. Our aim was to determine the resistance frequency over a period of 6 years to describe resistance phenotypes and to analyze underlying resistance mutations and trends.

## Methods

Five University Medical Centers (UMCs) participated in the surveillance network, including Leiden UMC (Leiden), Erasmus Medical Center (Rotterdam), Amsterdam UMC, VU Medical Center (Amsterdam), UMC Groningen (Groningen), and Radboud UMC (Nijmegen). The geographic regions include the west of the Netherlands (Rotterdam, Leiden, and Amsterdam), which is the most heavily populated region; the north (Groningen); and the east (Nijmegen). The centers were asked to screen *A. fumigatus* isolates cultured from clinical specimens by using an agar-based screening test (VIPcheck; MediaProducts, https://www.mediaproductsbv.nl). VIPcheck contains 3 agar wells supplemented with itraconazole, voriconazole, and posaconazole, and a growth control well (*11*). *A. fumigatus* colonies from the primary culture were inoculated on the 4-wells plate, incubated for up to 48 hours, and inspected for growth. If an isolate grew on any of the azole-containing wells, the isolate was sent anonymously to Radboud UMC for MIC testing and genotypic characterization. MIC testing was performed according to the European Committee on Antimicrobial Susceptibility Testing (EUCAST) broth microdilution reference method (*12*–*14*) for amphotericin B (AmB), itraconazole, voriconazole, posaconazole, and isavuconazole (added in 2015 after the drug was clinically licensed). Azole resistance was defined as resistance to >1 azole drug, according to EUCAST clinical breakpoints (itraconazole, >2 mg/L; voriconazole, >2 mg/L; posaconazole, >0.25 mg/L, and isavuconazole, >1 mg/L). EUCAST broth microdilution plates were made at Radboud UMC in batches of 250 96-well plates and complied with the recommended quality control standards (*11*–*13*). For *A. fumigatus* isolates with a confirmed azole-resistant phenotype, the full *Cyp51A* gene was analyzed by PCR amplification and sequencing (*7*). The Cyp51A sequence (GenBank accession no. AF338659) was used for mutation analysis. A spore suspension of all isolates was stored at −80**°**C in 10% glycerol.

Results of phenotypic testing were sent to the surveillance laboratories as soon as these were available. Analysis of the resistance genotypes was batched, and once a year each center received a list of isolates with resistance genotype and phenotype. The list of isolates was checked by the centers, who also provided the number of *A. fumigatus* culture-positive patients who had been screened for azole resistance during the year and the number of patients who harbored an azole-resistant isolate. Clinical information regarding underlying disease and classification of *Aspergillus* disease was not collected. Data on *A. fumigatus* resistance epidemiology are reported and published annually (*15*).

We calculated mean MICs with 95% CIs with GraphPad Prism 5.03 (https://www.graphpad.com). For calculations, we recoded MICs >16 mg/L as 32 mg/L. We calculated statistical tests on differences in MIC distributions by using Kruskal-Wallis test and tests on differences in classification according to clinical breakpoints by using the Fisher exact test.

## Results

### General Epidemiology

During 2013–2018, *A. fumigatus* isolates from 4,518 culture-positive patients were screened for the presence of azole-resistance. In 1 center, prospective screening was not performed in 2015, but only selected isolates from 22 patients were analyzed (Table 1). Therefore, we excluded these patients from calculation of the azole-resistance frequency, leaving 4,496 patients who had been screened for azole resistance. In total, 508 patients (11%) harbored an azole-resistant *A. fumigatus* isolate. Over the 6-year period, the overall resistance frequency increased from 7.6% (95% CI 5.9%–9.8%) in 2013 (58/760 patients) to 14.7% (95% CI 12.3%–17.4%) in 2018 (112/764 patients; p = 0.0001).

**Table 1:** Number of *Aspergillus fumigatus* culture-positive patients screened for azole resistance and azole resistance frequency in clinical *A. fumigatus* isolates in 5 University Medical Centers in the Netherlands*

Surveillance center	No. resistant/no. screened (%)					
	2013	2014	2015	2016	2017	2018
Erasmus MC, Rotterdam	10/231 (4.3)	10/265 (3.8)	7/22 (31.8)†	24/186 (12.9)	19/147 (12.9)	17/129 (13.2)
LUMC, Leiden	19/99 (19.2)	15/113 (13.3)	23/141 (16.3)	18/88 (20.5)	27/114 (23.7)	25/120 (20.8)
Radboud UMC, Nijmegen	6/123 (4.9)	7/143 (4.9)	12/145 (8.3)	20/210 (9.5)	21/198 (10.6)	23/196 (11.7)
UMCG, Groningen	16/194 (8.2)	18/191 (9.4)	15/225 (6.7)	26/215 (12.1)	35/240 (14.6)	34/238 (14.3)
VUMC, Amsterdam	8/113 (7.1)	9/104 (8.7)	14/89 (15.7)	13/85 (15.3)	12/75 (16.0)	13/81 (16.0)
Total	58/760 (7.6)	59/814 (7.2)	64/600 (10.7)‡	101/784 (12.9)	114/774 (14.7)	112/764 (14.7)

*MC, medical center; UMC, university medical center. †Only a limited number of patients was screened for azole resistance. ‡Calculation of resistance frequency did not include the cases of Erasmus MC.

### Triazole-Resistance Genotypes

**Figure 1:**
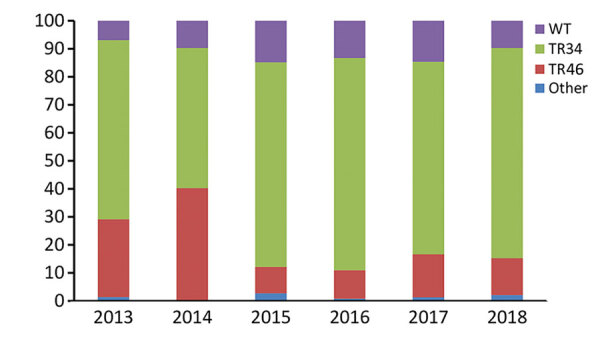
Distribution of *Cyp51A*-mediated resistance mutations in *Aspergillus fumigatus*, as observed in a national multicenter surveillance program in the Netherlands, 2013–2018. WT, wildtype *Cyp51A*; TR34, TR_34_/L98H; TR46, TR_46_/Y121F.

Overall, 640 *A. fumigatus* isolates (obtained from 508 patients) exhibited phenotypical resistance for >1 triazole. TR_34_/L98H was the most frequently observed resistance mechanism and was present in 445 (69%) azole-resistant *A. fumigatus* isolates, whereas TR_46_/Y121F/T289A was present in 111 (17%) isolates. Of 445 TR_34_/L98H isolates, 24 had >1 additional polymorphisms in the *Cyp51A* gene (F495I, n = 9; Q259H, n = 5; S297T, n = 4; D262N, n = 1; N326H, n = 1; P337L, n = 1; Y341H, n = 1; I364V, n = 1; G328A, n = 1; and L399V, n = 1). In addition, 8 TR_34_/L98H isolates harbored a T67G substitution in the gene promotor region, which has not been associated with azole resistance. TR-mediated resistance mutations are associated with resistance selection in the environment, which thus accounted for 86% of resistance mutations. In 76 azole-resistant isolates (12%), no mutations were found in the *Cyp51A* gene, indicating that other, yet uncharacterized, resistance mechanisms might be present. Over the 6-year observation period, no significant trends in the distribution of resistance mutations was observed (Figure 1).

### Triazole-Resistance Phenotypes

Resistance mutations most commonly affected the activity of all 4 mold-active azoles. Among the 640 azole-resistant *A. fumigatus* isolates, 413 (65%) isolates exhibited a panazole-resistant phenotype, 51 (8%) a multiazole-resistant phenotype, and 176 (28%) resistance to a single azole.

Because voriconazole is the treatment of choice for invasive aspergillosis, the azole resistance phenotypes were categorized according to voriconazole clinical breakpoints. Overall, 498 (77.8%) *A. fumigatus* isolates were voriconazole-resistant (Table 2). Although most voriconazole-resistant isolates exhibited a panazole-resistant phenotype, 50 (10%) voriconazole-resistant isolates were itraconazole-susceptible, of which 8 were also susceptible to posaconazole. Isolated posaconazole susceptibility in voriconazole-resistant isolates was not observed. The underlying resistance mutations detected in these 50 isolates included TR_46_/Y121F/T289A (35 isolates) and G448S (3 isolates), whereas *Cyp51A* mutations were not detected in 12 isolates. All voriconazole-resistant isolates were also resistant to isavuconazole.

**Table 2:** Resistance profiles of 640 azole-resistant *Aspergillus fumigatus* isolates classified according to voriconazole clinical breakpoints in a national multicenter surveillance program in the Netherlands, 2013–2018*

Voriconazole classification (no. isolates)	No. (%) isolates		
	Itraconazole	Posaconazole	Isavuconazole
Voriconazole-susceptible (18)			
Susceptible	0	1 (5.6)	6/16 (37.5)
Intermediate	1 (5.6)	2 (11.1)	NA
Resistant	17 (94.4)	15 (83.3)	10/16 (62.5)
Voriconazole-intermediate (124)			
Susceptible	0	4 (3.2)	2/121 (1.7)
Intermediate	6 (4.8)	19 (15.3)	NA
Resistant	118 (95.2)	101 (81.5)	119/121 (98.3)
Voriconazole-resistant (498)			
Susceptible	50 (10)	8 (1.6)	0/396 (0)
Intermediate	25 (5)	28 (5.6)	NA
Resistant	423 (85)	462 (92.8)	396/396 (100)

*Isavuconazole was not measured before 2014; therefore, denominator is different in comparison with itraconazole and posaconazole. NA, not applicable (no intermediate susceptibility category defined for isavuconazole).

In 124 (19%) *A. fumigatus* isolates, a voriconazole MIC of 2 mg/L was measured (Table 2); of these, 120 (97%) were resistant against either itraconazole, posaconazole, or both. The 4 remaining isolates were itraconazole- and posaconazole-susceptible but isavuconazole-resistant. Three of these isolates harbored the TR_34_/L98H mutation, and in the fourth isolate, no *Cyp51A* mutations were found. An underlying resistance mutation was detected in 99 (80%) of the 124 isolates with voriconazole-intermediate susceptibility.

Overall, 18 (3%) azole-resistant isolates were phenotypically voriconazole-susceptible, although these isolates were resistant to either itraconazole, posaconazole, or both (Table 2). Isavuconazole MICs were available for 16 phenotypically voriconazole-susceptible isolates, and isavuconazole resistance was found in 10 (63%) of these, with a mean isavuconazole MIC of 11 mg/L (range 2 to >16 mg/L). Underlying *Cyp51A*-mediated resistance mutations in these 10 isavuconazole-resistant, voriconazole-susceptible isolates included 6 isolates with a TR_34_/L98H genotype, although in 4 isolates no *Cyp51A* mutations were detected.

**Figure 2:**
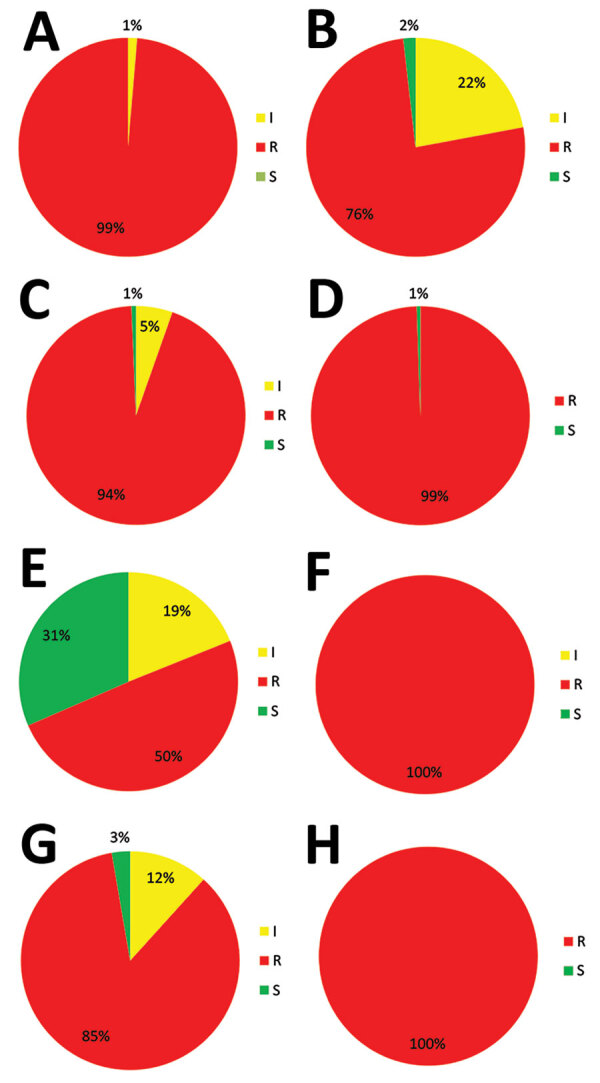
Triazole-resistance classification in 555 *Aspergillus fumigatus* isolates harboring TR_34_/L98H and TR_46_/Y121F/T289A resistance mutations, as observed in a national multicenter surveillance program in the Netherlands, 2013–2018. A) Itraconazole TR_34_/L98H. B) Voriconazole TR_34_/L98H. C) Posaconazole TR_34_/L98H. D) Isavuconazole TR_34_/L98H. E) Itraconazole TR_46_/Y121F/T289A. F) Voriconazole, TR_46_/Y121F/T289A. G) Posaconazole TR_46_/Y121F/T289A. H) Isavuconazole TR_46_/Y121F/T289A. I, intermediate; R, resistant; S, susceptible.

TR_34_/L98H is associated with high resistance to itraconazole, which was the case in 438 of 445 (98%) isolates, although TR_46_/Y121F/T289A is associated with high voriconazole resistance, which was found in all 111 TR_46_/Y121F/T289A isolates (Figure 2). Thirty-five of 111 (32%**)** TR_46_/Y121F/T289A isolates were susceptible to itraconazole, although 3 of these isolates were also susceptible to posaconazole.

### Trends in Voriconazole Resistance

**Figure 3:**
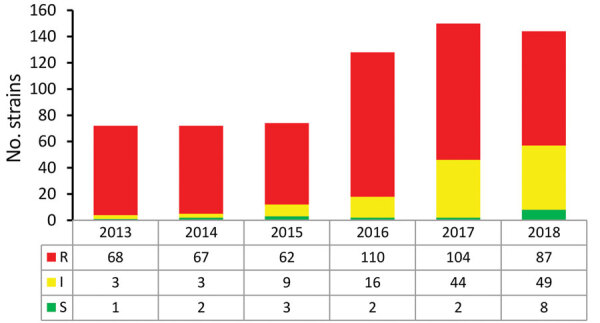
Trends in voriconazole-susceptibility classification of 640 *Aspergillus fumigatus* isolates, by year, as observed in a national multicenter surveillance program in the Netherlands, 2013–2018. I, intermediate; R, resistant; S, susceptible.

**Figure 4:**
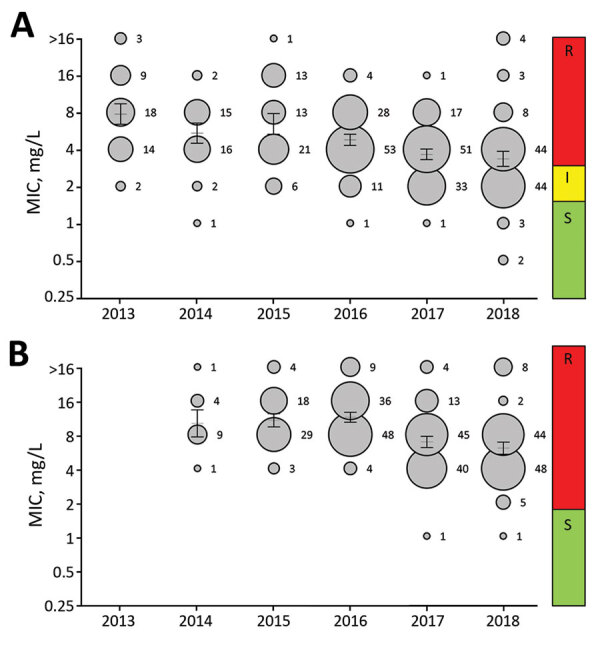
Trends in voriconazole (A) and isavuconazole (B) MIC distributions in *Aspergillus fumigatus* harboring TR_34_/L98H, as observed in a national multicenter surveillance program in the Netherlands, 2013–2018. MIC distribution is displayed as a bubble graph for each year, where the diameter corresponds with the number of isolates with the corresponding MIC. The number of isolates is presented for each MIC. Mean MIC with 95% CIs are plotted for each year as a line with error bars. The clinical interpretation is shown on the right of the diagram. I, intermediate; R, resistant; S, susceptible.

In 2013, voriconazole resistance was detected in 94% (68/72) of azole-resistant *A. fumigatus* isolates, but in 2018 voriconazole resistance was detected for only 60% (87/144) azole-resistant *A. fumigatus* isolates (p = 0.0001) (Figure 3). The trend toward lower voriconazole MICs was not attributable to a shift in resistance mutations but was apparent mainly in isolates harboring TR_34_/L98H (Figure 4, panel A). Voriconazole MIC distributions were significantly different when 2013 (mean voriconazole MIC 8 mg/L) was compared with 2018 (mean voriconazole MIC 2 mg/L) (p<0.001). In 2013, 2 (4%) of 46 TR_34_/L98H isolates were not classified as voriconazole-resistant, whereas 49 (4%) of 108 (45%) TR_34_/L98H isolates exhibited a voriconazole-nonresistant phenotype in 2018 (p = 0.0001), of which most exhibited a MIC of 2 mg/L (intermediate). Because a F495I mutation in TR_34_/L98H isolates was shown to be associated with reduced resistance to voriconazole (*16*), all 106 voriconazole-nonresistant TR_34_/L98H isolates were checked for the presence of this mutation. Only 8 TR_34_/L98H isolates were found to harbor the F495I mutation.

Because MIC testing was performed on receipt of the isolate over a 6-year period and thus involved various batches of MIC plates, all 46 TR_34_/L98H isolates from 2013 and 106 isolates (2 isolates were not available) from 2018 were retested for voriconazole and isavuconazole by using a single batch of MIC plates. Retesting confirmed the initial observation showing a 45% lower voriconazole-resistance frequency among TR_34_/L98H isolates in 2018 compared with 2013 (46/46 in 2013 vs. 58/106 in 2018; p = 0.0001) (Appendix Figure). In 2013, the overall azole-resistance frequency was 7.6% at the patient level and voriconazole-resistance frequency was 7.2%, whereas in 2018 the azole-resistance frequency was 14.7% compared with 8.8% voriconazole resistance (Table 1 [estimated voriconazole resistance frequencies not shown]).

We observed a similar decreasing trend of MICs for isavuconazole (Figure 4, panel B; Appendix Figure). However, the decrease in MICs did not result in an increase of susceptible isolates because all but 2 TR_34_/L98H isolates remained isavuconazole-resistant based on the EUCAST breakpoint. The isavuconazole MIC distributions were significantly different when the distribution of 2015 was compared with that of 2018 (p<0.001). No trends in phenotype changes were observed for itraconazole and posaconazole.

## Discussion

Our resistance surveillance showed an increasing frequency of azole resistance in clinical *A. fumigatus* isolates during 2013–2018 in the Netherlands. Although the resistance frequency varied between the 5 UMCs, the increasing trend was observed in all centers. In 2017 and 2018, the azole-resistance frequency exceeded 10% in all centers, a threshold above which experts recommend reconsidering the use of first-line voriconazole monotherapy (*17*,*18*). Alternative regimens that cover resistance include voriconazole/isavuconazole in combination with liposomal-AmB or an echinocandin, or monotherapy with liposomal-AmB. As such, the Netherlands national guideline was revised and now recommends combination therapy in patients with suspected invasive aspergillosis, in particular for critically ill patients or when azole resistance cannot be excluded (*19*).

How the 10% resistance threshold should be determined remains unclear (*17*,*18*). Our study indicated that despite the increasing azole-resistance frequency, the resistance frequency of voriconazole remained below this threshold (8.8% in 2018). This observation warrants the question of which threshold (i.e., azole resistance or voriconazole resistance) should be used to guide decisions regarding primary treatment choices. The trend toward lower voriconazole MICs was observed mainly in TR_34_/L98H isolates, whereas most nonresistant isolates exhibited a voriconazole MIC of 2 mg/L (intermediate). The optimal management of voriconazole-intermediate invasive aspergillosis remains unclear, but an increased failure rate is anticipated in patients treated with voriconazole monotherapy at the standard dose. An increased voriconazole trough level target of 2–6 mg/L is recommended or combining voriconazole with an echinocandin or liposomal-AmB monotherapy (*18*). Furthermore, there is currently insufficient evidence that infection caused by azole-susceptible or intermediate susceptible *A. fumigatus* isolates that harbor a resistance mutation can safely be treated with azole monotherapy.

Resistance was highly dominated by environmental resistance mutations, such as TR_34_/L98H and TR_46_/Y121F/T289A. Indeed, a recent study identified sites at high risk for resistance selection in the environment, referred to as hotspots (*20*). Particularly, stockpiling of decaying plant waste containing azole fungicide residues was found to harbor high numbers of resistant *A. fumigatus*, and isolates with identical resistance mutations to those recovered from clinical specimens were cultured from these hotspots (*20*,*21*). The observations support a strong link between environmental resistance selection and azole-resistant disease in humans. The increasing trend in azole-resistance frequency and the emergence of new resistance mutations indicate that the current use of azole fungicides is not sustainable and over time the medical use of azoles will be further threatened. Given the limited alternative treatment options for *Aspergillus* diseases, further research aimed at reducing the burden of azole resistance in the environment is urgently needed.

An important question is how the changing voriconazole phenotype in TR_34_/L98H isolates can be explained. One possibility is that TR_34_/L98H isolates harbor additional mechanisms or compensatory mutations that affect the overall azole-resistance phenotype. Additional changes in peptide sequence of the 14-α-sterol demethylase enzyme have been reported in TR_34_/L98H isolates, such as F495I, which confers resistance to imidazole (*16*). Recombination experiments showed that recombinants with S297T/F495I in the TR_34_/L98H background conferred high resistance to imidazole but also produced lower voriconazole MICs compared with the TR_34_/L98H parent strain (*16*). Because TR_34_ isolates originate in the environment through exposure to azole fungicides, changes in azole fungicide exposures could prompt changes in resistance phenotypes. However, only 9 isolates with an additional F495I mutation in the *Cyp51A* gene were found, of which 8 were voriconazole-susceptible, indicating that other or multiple factors might have contributed to the observed phenotype change.

Another possibility is the presence of other resistance mechanisms in TR_34_/L98H isolates. Recent studies analyzing transcriptional control mechanisms of *Cyp51A*, have identified transcription factors, such as ABC transporter regulator, which was found to regulate many different processes involved in drug resistance, metabolism, and virulence (*22*). Furthermore, other steps in the ergosterol biosynthesis might be affected, for example, through mutations in the 3-hydroxy-3-methyl-glutaryl-coenzyme A reductase-encoding gene (*hmg1*) (*23*). Mutations in the *hmg1* gene resulting in peptide sequence changes were found to be frequent in TR_34_/L98H isolates, possibly affecting the azole phenotype (*23*). These recent insights indicate that the resistance phenotype is likely to be multifactorial and that more research is needed to characterize possible mechanisms that explain the observed variation in TR_34_/L98H phenotypes.

The main clinical implication of the observed phenotypic variation in TR_34_/L98H relates to direct detection of resistance mutations through PCR tests. Several commercial PCR tests are available that enable detection of TR_34_/L98H and TR_46_/Y121F/T289A directly in clinical specimens (*24*,*25*), which are used to guide selection of antifungal drugs. However, this approach can only be used if the resistance genotype predicts the azole phenotype. Although TR_46_/Y121F/T289A mutation detection is uniformly associated with resistance to voriconazole, the wide spectrum of voriconazole MICs in TR_34_/L98H isolates hampers the use of direct detection of this mutation because voriconazole therapy might be withheld in cases of voriconazole-susceptible infection. However, as stated previously, the efficacy of voriconazole in voriconazole-susceptible and voriconazole-intermediate TR_34_/L98H infection would need to be investigated before any treatment recommendations involving azole monotherapy can be implemented.

Our resistance surveillance had several limitations. All *A. fumigatus* isolates cultured from clinical specimens were screened for azole resistance, involving diverse patient groups and isolates not regarded clinically relevant. Including all isolates has the advantage of collecting a meaningful number of isolates, but the resistance frequency might not be representative for specific patient groups such as those with invasive aspergillosis. Furthermore, within hospitals the resistance frequency might vary between years (*26*), thus further complicating establishing a meaningful (local) resistance frequency. Another limitation was the lack of clinical information and *Aspergillus* disease classification. Such information would be more suitable to guide treatment decisions and local antifungal guidelines, but gathering the data is laborious, given that all aspergillosis cases need to be identified and classified. Various cohort studies have been performed in the Netherlands showing differences in resistance rates between patient groups. A resistance frequency of 26% (10 of 38 patients) was reported in *A. fumigatus* culture-positive intensive-care unit patients in a single hospital (*27*) and 29% (4 of 14 patients) in a national study of intensive-care unit patients with influenza-associated aspergillosis (*2*). In contrast, a low resistance frequency was reported in a 5 year single-center cohort of patients with hematologic malignancy (*28*), but the culture-positivity rate was only 6% and thus, in most patients, the presence of resistance remained unknown. To date, only few studies have determined the frequency of resistance mutations in culture-negative patients. In 1 study, the rate was found to be similar to that observed in culture-positive patients (*29*), although higher resistance rates were reported in culture-negative patients in comparison with the rates found by culture in another study on patients with chronic rather than acute invasive aspergillosis (*30*). Differences could be explained in part by coincidence because the number of cases in these single-center studies is small. Another explanation might be the number of *A. fumigatus* colonies that were tested for resistance, given that clinical cultures might contain azole-susceptible and azole-resistant colonies in cases of mixed infection (*31*,*32*).

In conclusion, azole resistance in *A. fumigatus* has been reported worldwide and provides major challenges regarding management of invasive aspergillosis and other *Aspergillus* diseases (*9*,*32*). Nevertheless, *A. fumigatus* is not included in global action plans to combat antimicrobial resistance (*33*), and no international surveillance programs monitor resistance in *A. fumigatus.* As a consequence, the presence and frequency of azole resistance remains unknown in most countries (*34*,*35*). Despite the challenges we face in performing resistance surveillance in *A. fumigatus*, our national surveillance has proved important to guide the national treatment guideline and provided insights in trends in resistance genotypes and phenotypes. Furthermore, continued surveillance will help to monitor effects of interventions aimed at reducing the resistance burden in the environment. We believe that global *A. fumigatus* resistance surveillance programs are warranted and should be implemented in initiatives to combat antimicrobial resistance.

Appendix Additional information about paradoxal trends in azole-resistant *Aspergillus fumigatus* in a national multicenter surveillance program, 2013–2018.
