# Supplementary material for: Paradoxal Trends in Azole-Resistant Aspergillus fumigatus in a National Multicenter Surveillance Program, the Netherlands, 2013–2018
Source: Emerg Infect Dis. 2020 Jul;26(7):1447–55. doi: 10.3201/eid2607.200088 (PMC7323544; doi:10.3201/eid2607.200088)
Supplement: Appendix — Additional information about paradoxal trends in azole-resistant Aspergillus fumigatus in a national multicenter surveillance program, 2013–2018. [file 20-0088-Techapp-s1.pdf]

Article DOI: https://doi.org/10.3201/eid2607.200088

Paradoxal Trends in Azole-Resistant *Aspergillus fumigatus* in a National Multicenter Surveillance Program, the Netherlands, 2013–2018

Appendix

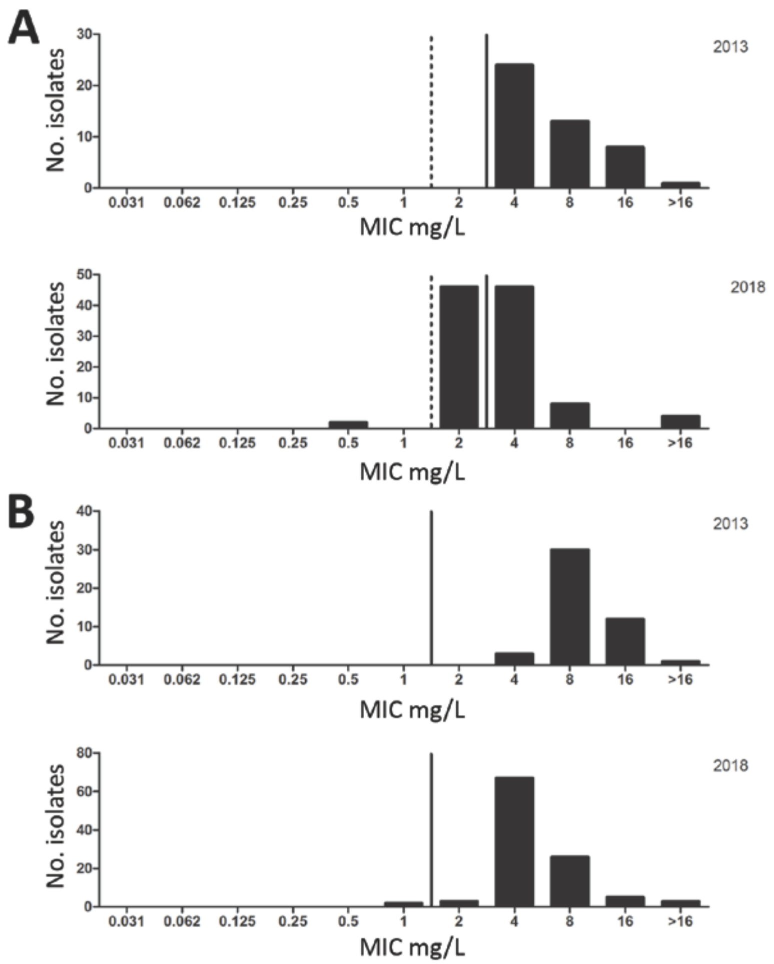

Appendix Figure. Voriconazole and isavuconazole MIC distributions of *A. fumigatus* isolates cultured in 2013 and 2018 harboring TR_{34}/L98H retested in a single batch. MIC distributions of voriconazole and isavuconazole were significantly different when the MIC distributions of 2013 and 2018 (voriconazole), and 2015 and 2018 (isavuconazole) were compared (Kruskall Wallis $p<0.001$). Clinical breakpoints are indicated by vertical lines.
